# NDF/GLYR1 Promotes RNA Polymerase II Processivity via Pol II Binding and Nucleosome Destabilization

**DOI:** 10.3390/ijms26104874

**Published:** 2025-05-19

**Authors:** Ziwei Li, Jia Fei

**Affiliations:** 1 Department of Molecular Biology and Biochemistry, Rutgers University, Piscataway, NJ 08854, USA; ziwei.li@rutgers.edu; 2 Rutgers Cancer Institute of New Jersey, New Brunswick, NJ 08901, USA

**Keywords:** NDF, RNA polymerase II, transcription, elongation

## Abstract

The Nucleosome Destabilizing Factor (NDF) facilitates transcription through chromatin, but its precise mechanism remains incompletely understood. Here, we identify a critical region (amino acids 140–160) within NDF that specifically interacts with phosphorylated RPB1, the largest subunit of elongating RNA Polymerase II (Pol II). Mutations in this region disrupt Pol II interaction and impair Pol II elongation both in vitro and in cells, yet do not affect NDF’s ability to destabilize nucleosomes, establishing a functional separation between these two activities. Cellular studies reveal that NDF knockout cells display faster Pol II elongation rates but produce fewer nascent transcripts, demonstrating NDF’s primary role in maintaining transcriptional processivity throughout gene bodies. Our findings demonstrate that NDF uses distinct mechanisms to ensure productive transcription elongation rather than simply enhancing elongation speed, offering new insights into how transcription efficiency is maintained in chromatin.

## 1. Introduction

Transcription by eukaryotic Pol II is regulated at multiple steps, among which the transition from pausing into productive elongation has emerged as a critical control point [1,2,3]. After initiation, Pol II frequently pauses shortly downstream of promoters due to the concerted action of negative elongation factors [4]. In particular, the DRB sensitivity-inducing factor (DSIF) and negative elongation factor (NELF) associate with Pol II to induce a promoter-proximal pause [5,6]. This pausing mechanism allows for checkpoint regulation of early transcripts and is stabilized by NELF, which prevents premature termination of the paused Pol II [7,8,9]. Release of Pol II into productive elongation requires positive transcription elongation factor b (P-TEFb), a kinase complex that phosphorylates NELF, DSIF, and the Pol II C-terminal domain (CTD), thereby dissociating NELF and converting DSIF into a positive elongation factor [7]. Through this transition, Pol II enters a highly processive elongation phase.

Upon release from the paused state, Pol II encounters a subsequent significant obstacle: traversing chromatin architecture wherein nucleosomes function as structural impediments to transcriptional progression [10,11,12]. Notably, although chromatin-mediated Pol II pausing in vivo is predominantly detected near the +1 nucleosome position, with minimal evidence supporting nucleosome-induced pausing throughout gene bodies [13,14,15], isolated Pol II in vitro demonstrates an inability to transcribe through even individual nucleosomal structures [10,11,12]. This apparent discrepancy indicates the presence of cellular accessory factors that facilitate Pol II navigation through nucleosomal barriers during the transcription elongation phase [1,16,17].

This discrepancy between in vivo and in vitro observations has driven the identification and characterization of numerous elongation factors with diverse functions. Some factors, such as FACT (FAcilitates Chromatin Transcription), act as histone chaperones that destabilize nucleosomes by removing/binding to H2A-H2B dimers [18,19,20,21,22,23,24,25,26,27,28]. Others, like the ATP-dependent chromatin remodelers (e.g., SWI/SNF, RSC, CSB, and CHD1), use energy from ATP hydrolysis to slide or evict nucleosomes [29,30,31,32]. Additionally, factors such as TFIIS, PAF, Spt6, DSIF, and NELF, *p*-TEFb, and Mediator kinase module directly interact with Pol II to mediate RNA polymerase transcription activity through different mechanisms (for reviews, see [3,9,16,17,33,34,35,36]).

Among these factors, the Nucleosome-destabilizing Factor (NDF, also known as Glyr1, N-PAC, or NP60) has emerged with unique properties distinct from other elongation factors. NDF consists of an N-terminal PWWP domain, which can bind H3K36me3, and a C-terminal dehydrogenase-like domain that lacks detectable dehydrogenase activity [37]. The human homolog of NDF was identified through proteomics studies as a factor that preferentially binds to H3K36me3 histones and is present at high levels in all tested tissues in humans [38,39]. NDF is essential in stem cells and is frequently overexpressed in breast cancer [40]. In Drosophila, NDF was previously identified as an MSL (male-specific lethal) complex-interacting protein that plays a role in X-chromosome dosage compensation [41]. In yeast *S. cerevisiae*, the NDF-related protein is Pdp3 (yPdp3), which shares significant similarity (~25% identity, ~39% similarity) with the N-terminal region of human NDF but lacks the inactive dehydrogenase domain. yPdp3 localizes to gene bodies, stimulates transcription by yeast Pol II, indicating conserved transcriptional roles across species [42]. Additionally, NDF has been shown to interact with and boost the catalytic activity of the histone demethylase LSD1/KDM1A, suggesting a role in regulating histone methylation status and transcription [37,43].

In our previous studies, we showed that NDF exhibits ATP-independent nucleosome destabilization activity that enhances transcription by reducing the energy barrier for Pol II elongation [44]. In cells, we observed that NDF is specifically recruited to genes upon their activation [44]. Subsequent investigations demonstrated that NDF not only functions on chromatin templates but also interacts with Pol II to stimulate transcription on naked DNA [42], indicating that NDF possesses both chromatin-dependent and chromatin-independent mechanisms to promote transcriptional activity.

Despite our characterization of these novel activities of NDF, important mechanistic questions remain unanswered. First, the biophysical basis by which NDF destabilizes nucleosomes in the absence of ATP hydrolysis is not yet understood. Although it is well-established that NDF binds to H3K36me3 via its PWWP domain [45], the nature of its interactions with the remainder of the nucleosome core particle and the molecular forces driving its destabilization activity require further investigation. Second, the mechanism by which NDF stimulates Pol II elongation on non-chromatin templates is unknown. It also remains to be determined whether nucleosome disassembly and transcription stimulation are linked through a single mechanism or mediated by separate protein domains. Addressing these issues will greatly enhance our understanding of NDF’s role in transcriptional regulation through chromatin.

In this study, we identify and characterize a critical domain in NDF that specifically interacts with phosphorylated RNA Polymerase II and demonstrate that this interaction is functionally distinct from NDF’s nucleosome destabilization activity. Through biochemical, mutational, and cellular analyses, we show that NDF employs independent mechanisms to regulate chromatin accessibility and Pol II elongation activity. Intriguingly, NDF regulates transcription by affecting processivity rather than elongation speed, thereby ensuring robust transcript completion instead of merely accelerating polymerase velocity. The cooperation between these dual functions significantly enhances transcription through nucleosomal barriers. These findings provide mechanistic insights into how NDF operates as a multifaceted transcriptional coregulator facilitating Pol II navigation through chromatin.

## 2. Results

### 2.1. Identification of the Domain in NDF Required for Interaction with Pol II

Understanding the molecular mechanisms by which NDF stimulates Pol II transcription elongation requires detailed characterization of their interaction. Based on our previous findings that NDF directly enhances Pol II activity [42], we attempted to solve the cryo-EM structure of their complex. Despite employing various sample preparation strategies with NDF homologs from yeast (Pdp3), human (GLYR1/NDF), and Drosophila (CG4747), we could not detect NDF densities in the 2D reconstitution, although Pol II was well-resolved (Supplemental Figure S1A). Disorder prediction analysis using Dismeta [46] revealed extensive intrinsically disordered regions within NDF. We hypothesize that this conformational flexibility may contribute to the challenges encountered in structural studies, though this remains to be experimentally verified.

**Figure 1:**
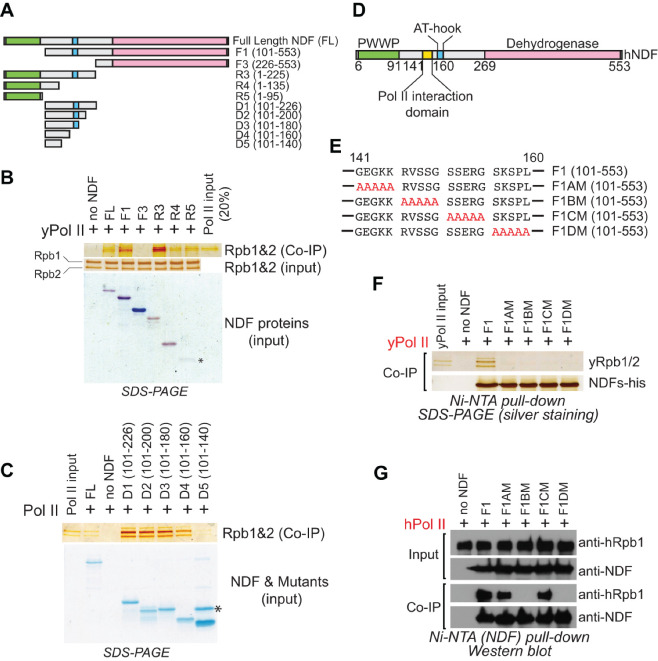
Identification and Characterization of the NDF Domain that Interacts with Pol II. (**A**) Schematic representation of full-length NDF and its truncated variants used for domain mapping of Pol II interaction. (**B**,**C**) Pull-down assays showing interactions between His6-tagged NDF truncation variants and untagged yeast Pol II. Proteins were captured using Ni-NTA resin, resolved by SDS-PAGE, and visualized by Coomassie blue or silver staining. Asterisk (*) in panel (**B**) indicates the R5 truncate, which is stained faintly in Coomassie blue staining. Asterisk (*) in panel (**C**) indicates a nonspecific band in the D5 protein prep. (**D**) Schematic summary of the NDF domain analysis showing the region between amino acids 140–160 required for Pol II interaction, as determined from refined mapping experiments in panels (**B**,**C**,**E**) Amino acid sequences of F1 (NDF fragment 1) alanine-scanning mutants. Consecutive blocks of five amino acids within the 140–160 region were substituted with alanines to generate mutants F1AM, F1BM, F1CM, and F1DM. (**F**) Pull-down assays demonstrating the interaction between His6-tagged F1 mutants and yeast Pol II. All four alanine substitution mutants abolished interaction with yeast Pol II, indicating the entire 140–160 region is critical for binding. (**G**) Interaction study between His6-tagged F1 mutants and human Pol II (FLAG-tagged) using a Ni-NTA pull-down assay. Proteins were separated on a 4–20% SDS-PAGE and detected by Western blotting.

Given these challenges with direct structural approaches, we shifted to systematic biochemical mapping to identify the interaction domains. We generated a set of NDF truncation constructs spanning the entire protein length, including both N-terminal and C-terminal truncates (Figure 1A). These constructs were engineered with hexahistidine tags to facilitate purification and binding assays, were expressed in *E. coli*, and were purified as recombinant proteins.

Using these purified truncation variants, we performed in vitro pull-down assays with purified yeast Pol II using stringent salt conditions (200 mM NaCl) to minimize non-specific interactions while enabling the detection of genuine binding. These experiments revealed two distinct domains within human NDF that mediate binding to Pol II. The first interaction domain is located within the N-terminal PWWP domain (amino acids 1–91, Figure 1B). PWWP domains are well-known modules in chromatin-associated proteins that typically recognize histone modifications, especially H3K36me3. However, these domains generally feature positively charged surfaces that facilitate nucleic acid and histone binding (for example, [47,48,49]). Due to this characteristic, we were concerned that the PWWP–Pol II interaction observed in vitro might result from non-specific electrostatic interactions rather than reflecting a genuine biological function. Further attempts to map the minimal interaction region within the PWWP domain suggested that amino acids 60–95 might be critical for Pol II binding (Supplementary Figure S1B). However, we interpret these truncation results with caution, as partial truncations may disrupt the compact folding of the PWWP domain, potentially exposing regions that are normally buried and thus generating artifactual interactions. Although our pull-down assays confirmed a direct interaction between the PWWP domain and Pol II, we chose to focus subsequent functional analyses on the second interaction domain, which exhibited more specific binding characteristics as detailed below.

This second, more significant interaction region resides within the central portion of NDF, spanning amino acids 101–226 (Figure 1B). Interestingly, this area aligns with a mostly disordered part of the protein, matching our earlier computational predictions. To map this interaction more precisely, we generated smaller truncation constructs within this region and performed additional pull-down assays. These experiments allowed us to narrow down the key interaction site to a shorter segment between amino acids 140 and 160 (Figure 1C,D).

To determine which residues within the 140–160 region are critical for interaction, we used alanine scanning mutagenesis. We systematically replaced five consecutive amino acids with alanines, creating four distinct mutant constructs (Figure 1E). These mutations were generated in a PWWP-less NDF truncation background (NDF_F1) to avoid potential interference from interactions mediated by the PWWP domain. Pull-down assays revealed that all four alanine-scanning mutants completely lost the ability to interact with yeast Pol II (Figure 1E,F). These results clearly indicate that the entire 140–160 segment forms an essential interaction surface with Pol II.

Intriguingly, when we tested these same mutants with human Pol II (hPol II), we observed a distinct interaction pattern. Only the F1BM (residues 146–150) and F1DM (residues 156–160) mutants lost their ability to interact with human Pol II, while F1AM and F1CM retained binding capability (Figure 1E,G). Given that yeast and human Pol II share a highly conserved core but differ significantly in their surface residues [50], this differential binding profile likely reflects species-specific adaptations in the NDF-Pol II interface. Taken together, our results indicate that the NDF-Pol II interaction is mediated through a defined, conserved segment within the central disordered domain of NDF.

### 2.2. NDF Preferentially Interacts with Hyperphosphorylated Rpb1

To better understand how NDF stimulates Pol II, we sought to identify the Pol II subunit responsible for the interaction. Pol II is a complex enzyme consisting of 12 subunits (Rpb1–Rpb12), with Rpb1 being the largest and containing both the catalytic site and the regulatory C-terminal domain (CTD) [50]. Mapping interactions using conventional co-immunoprecipitation approaches with recombinant Pol II subunits is challenging because these subunits typically become insoluble when expressed individually in *E. coli* [51].

To overcome this challenge, we used proximity-dependent biotinylation (BioID [52]), employing the promiscuous biotin ligase BirA*, to identify Pol II subunits closely interacting with NDF. BioID is effective in mapping protein-protein interactions because BirA* biotinylates proteins located within roughly 10 nm [53], providing a snapshot of the immediate protein environment. We generated and purified a BirA*-hNDF fusion protein.

**Figure 2:**
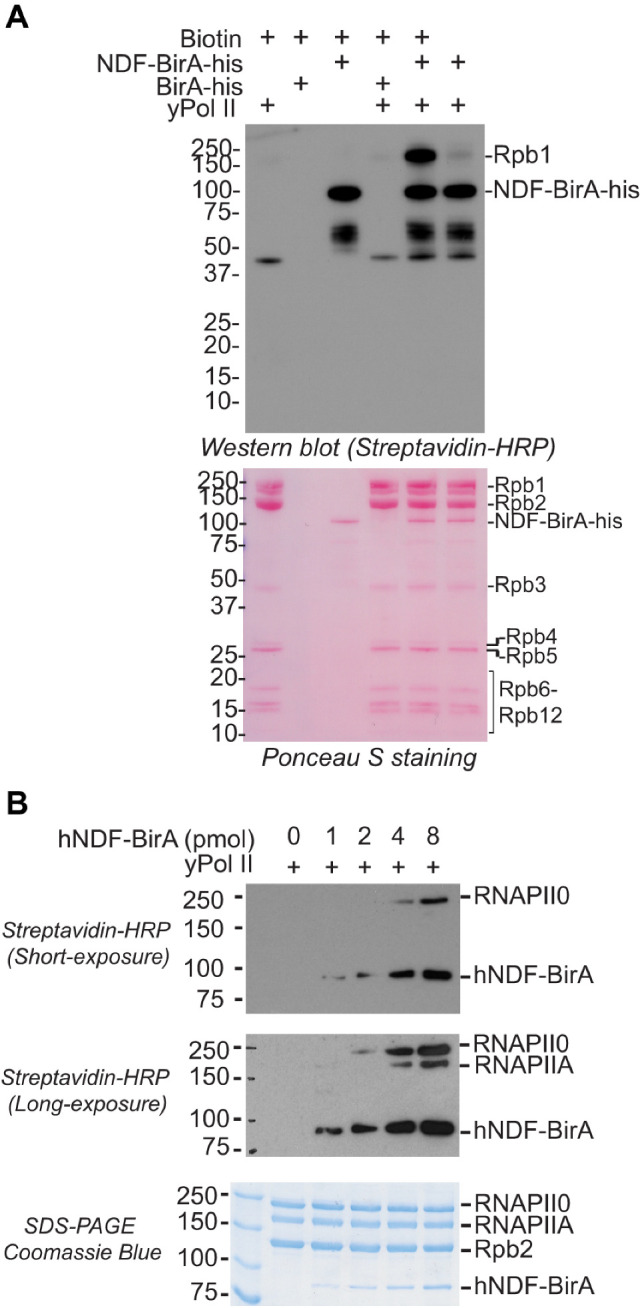
NDF Preferentially Interacts with the Hyperphosphorylated Rpb1 Subunit of Pol II. (**A**) Proximity-dependent biotinylation assay showing that BirA*-tagged hNDF specifically biotinylates the Rpb1 subunit of yeast Pol II. In vitro biotinylation experiments were performed with the indicated components. Biotinylated proteins were detected using streptavidin-HRP (upper panel), and protein transfer efficiency was evaluated by Ponceau S staining (lower panel). This assay reveals that NDF positions itself in close proximity to Rpb1 during its interaction with Pol II. (**B**) Dose-dependent biotinylation of yeast Pol II (25 pmol) with increasing amounts of hNDF-BirA* (0, 1, 2, 4, and 8 pmol). Biotinylated proteins were detected by streptavidin-HRP with short exposure (upper panel) and longer exposure (middle panel). Total protein levels were assessed by Coomassie Blue staining (lower panel).

We incubated purified BirA*-hNDF with yeast Pol II in the presence or absence of biotin and analyzed biotinylation patterns by SDS-PAGE and streptavidin-HRP western blotting. Notably, BirA*-hNDF specifically biotinylated only the Rpb1 subunit, with no detectable labeling of other Pol II subunits (Figure 2A). This result strongly suggests that NDF directly interacts with Rpb1 and positions itself near this critical catalytic subunit during its interaction with Pol II.

Further analysis showed that BirA*-hNDF preferentially biotinylated the hyperphosphorylated form of Rpb1 (subunit IIo, RNAPIIO) over the hypophosphorylated form (subunit IIa, RNAPIIA) (Figure 2B). Phosphorylation of the Rpb1 CTD is a critical regulatory step during transcription, with RNAPIIA predominantly involved in pre-initiation and initiation phases, and RNAPIIO marking active elongation complexes [54,55]. The selective interaction of NDF with RNAPIIO strongly indicates its specific role during transcription elongation rather than initiation. This observation is consistent with our previous ChIP-seq data, showing NDF enrichment in gene bodies rather than promoters, and aligns with the recruitment of NDF to actively transcribed genes upon activation [44].

### 2.3. Specific Interaction Domain of NDF Is Required for Stimulating Transcription by Pol II In Vitro

**Figure 3:**
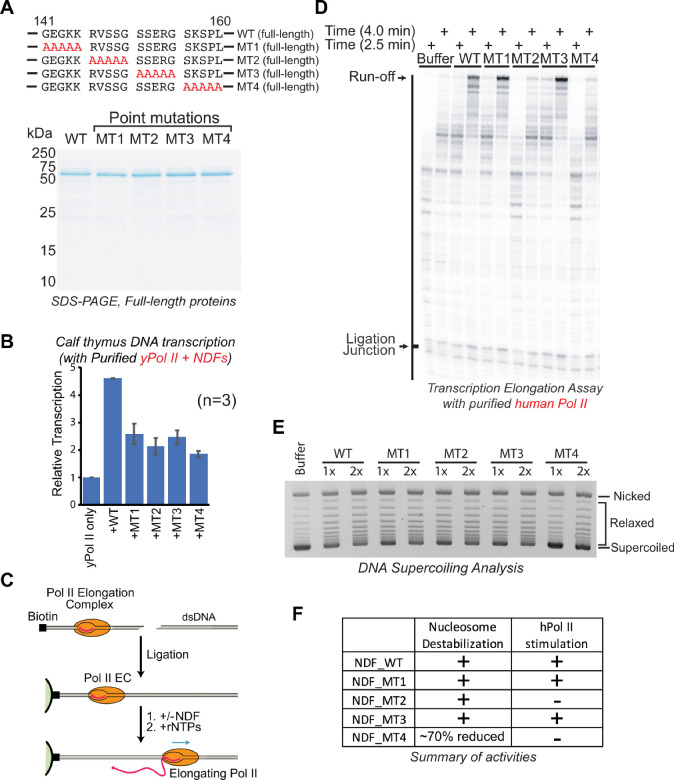
The Pol II interaction domain of NDF is required for stimulating transcription but is functionally distinct from nucleosome destabilization activity. (**A**) Amino acid sequences of various full-length hNDF mutants with indicated Alanine substitution (upper panel), and the SDS-PAGE analysis of purified full-length NDF and its mutants (lower panel). (**B**) Quantitative analysis of transcription stimulation activity using a calf thymus DNA template. Reactions containing [α-^32^P]CTP were performed with equal amounts of wild-type hNDF or mutant proteins, with transcriptional activity measured by radioactive CTP incorporation. All mutants show reduced transcription stimulation activity (~50% of wild-type), correlating with their reduced Pol II binding capacity. Error bars represent the standard deviation from three experiments. (**C**) Diagram of the transcription elongation assay with purified Pol II elongation complexes and a defined DNA template. (**D**) Transcription elongation reactions with purified human Pol II and the *Xenopus borealis* 5S rDNA template. Reactions were performed in the presence or absence of purified full-length hNDF and mutants for the indicated times. (**E**) Supercoiling assay performed using chromatin reconstituted with plasmid DNA and purified core histones by salt dialysis in the presence of topoisomerase I (Topo I) and different concentrations (0.05 and 0.1 µM) of hNDF and its mutants. Following deproteinization, samples were analyzed by 0.8% agarose gel electrophoresis, and DNA was visualized by ethidium bromide staining. Quantification of nucleosome destabilization activity shows that MT1, MT2, and MT3 retain similar activity to wild-type NDF (1.4×, 1.3×, and 1.3×, respectively), while MT4 exhibits significantly reduced activity (0.2× at 0.05 µM and 0.68× at 0.1 µM), relative to wild-type NDF (set as 1.0). (**F**) Summary of the activities of hNDF and its mutants. MT4 exhibits significantly reduced activity (71% reduction at 0.05 µM).

Having identified the specific NDF domain mediating the interaction with Pol II, we next asked whether this interaction is essential for the transcription-stimulating activity of NDF. To address this, we purified alanine-mutagenized full-length hNDF mutants (MT1, MT2, MT3, and MT4, corresponding to mutations in PWWP-less mutants F1AM, F1BM, F1CM, and F1DM, respectively) (Figure 3A) and tested their transcriptional stimulation using calf thymus DNA and yeast Pol II (Figure 3B).

In this assay, transcription initiates non-specifically at nicks, gaps, or DNA ends of purified calf thymus genomic DNA, and transcriptional activity was measured by incorporation of radiolabeled CTP into RNA. Samples were quantified by spotting onto filter disks that selectively bind nucleic acids. This method has been extensively utilized in early transcription studies, including the isolation of Pol II elongation factors such as TFIIS (S-II) [56,57]. We observed that although each alanine-mutant retained partial transcription-stimulation activity, their effectiveness was significantly reduced (by approximately 50%) compared to wild-type hNDF (Figure 3B). Importantly, this reduction in activity strongly correlated with the mutants’ reduced ability to bind Pol II (Figure 1F). These findings clearly demonstrate that direct interaction with Pol II is crucial for the full transcriptional stimulation activity of NDF. The residual activity observed suggests either weaker or transient interactions can partially stimulate transcription or that additional domains (such as the PWWP domain) also contribute to NDF’s function in vitro.

### 2.4. Specific Interaction Domain of NDF Is Required for Stimulating Transcription Elongation by Pol II

While the calf thymus DNA assay provided insights into the general transcription stimulation capability of NDF, it could not differentiate between initiation and elongation effects due to random transcription initiation sites. To specifically examine NDF’s function during elongation, we utilized a defined DNA template assay developed by Kashlev and colleagues [58,59,60], which aligns well with NDF’s chromatin enrichment across transcribed gene bodies. In this assay, a short 5′-labeled RNA primer was hybridized to the template DNA strand, purified hPol II was added to form an active elongation complex, and finally, a biotinylated non-template DNA strand was annealed to complete the complex (illustrated in Figure 3C, top left).

The elongation complex was immobilized onto magnetic beads, and downstream DNA sequences were ligated to the complex, enabling precise analysis of how hNDF and its mutants affect Pol II elongation through defined sequences. Importantly, our assembled elongation complex lacked other transcription factors such as DSIF or the NELF complex, allowing us to directly assess the specific effect of NDF on Pol II elongation activity. Transcription elongation was initiated by adding all four rNTPs.

Using this defined transcription system, we observed that hNDF mutants MT1 and MT3 retained the ability to stimulate transcription elongation, whereas mutants MT2 and MT4 lost this stimulatory effect, as evidenced by the levels of full-length runoff transcripts (Figure 3D). Full-length runoff transcripts indicate Pol II complexes successfully elongating through the entire DNA template, while shorter transcripts reflect paused or prematurely terminated transcription events.

This pattern of activity is consistent with our previous finding that the PWWP-less hNDF truncations F1BM and F1DM lost interaction with hPol II (Figure 1G). The mutations in MT2 (residues 146–150) and MT4 (residues 156–160) correspond to the same regions as in F1BM and F1DM, respectively, explaining their loss of stimulatory activity.

The selective loss of stimulatory activity in MT2 and MT4, but not in MT1 and MT3, despite all four mutants containing alterations within the 140–160 region, suggests that specific amino acid residues within this region are critical for the interaction with human Pol II and subsequent stimulation of transcription elongation.

Together, these findings indicate that the NDF’s stimulation of transcription elongation by Pol II is directly dependent on its physical interaction with Pol II, particularly with the Rpb1 subunit, and that this interaction relies on specific residues within the 140–160 region of NDF.

### 2.5. Distinct Domains of NDF Separately Mediate Nucleosome Destabilization and Pol II Stimulation

Previous studies have shown that NDF destabilizes nucleosomes [44]. To determine if the interaction domain of NDF is required for nucleosome destabilization, we assessed the mutants’ ability to disrupt nucleosome-mediated supercoiling of covalently closed circular plasmid DNA. Because nucleosome formation introduces negative supercoiling, nucleosome disassembly can be monitored by detecting the relaxation of this supercoiling. We reconstituted nucleosomes onto plasmid DNA via salt dialysis, then treated the chromatin with wild-type NDF or mutants in the presence of topoisomerase I. Analysis by agarose gel electrophoresis showed that mutants MT1, MT2, and MT3 retained nucleosome destabilization comparable to wild-type NDF, whereas MT4 exhibited a ~50% reduction (Figure 3E).

These results indicate that NDF possesses two distinct activities: nucleosome destabilization and Pol II stimulation. Importantly, these two activities appear to be mechanistically separable, as demonstrated by mutant MT2, which retained nucleosome destabilization activity but lost the ability to stimulate Pol II (Figure 3F). This clear functional separation supports the idea that NDF utilizes distinct mechanisms for its multiple roles in transcription elongation, though we cannot definitively conclude which specific domains are responsible for nucleosome destabilization based on the current data.

### 2.6. NDF Regulates Transcription Through Processivity Rather than Elongation Rate

While our findings demonstrate that NDF employs mechanistically distinct activities for nucleosome destabilization and Pol II stimulation, the functional consequences of these activities on overall transcription dynamics remained unclear. In our previous studies with HeLa cells, we observed that NDF knockout had minimal effects on mature mRNA levels as measured by RNA-seq and transcriptionally active Pol II distribution as measured by GRO-seq, which raised questions about NDF’s specific contribution to transcription elongation. To clarify this apparent discrepancy between NDF’s biochemical activities and cellular phenotypes, we investigated how NDF affects different aspects of transcription elongation in HeLa cells.

We employed a DRB-release protocol coupled with TT-seq (transient transcriptome sequencing) to distinguish between elongation speed/rate and processivity in wild-type and NDF knockout HeLa cells. By inhibiting the CDK9 subunit kinase activity of the P-TEFb complex with 5,6-dichloro-1-β-D-ribofuranosyl benzimidazole (DRB), we arrested Pol II at promoter-proximal sites while allowing already-engaged polymerases to complete transcription. After clearing all elongating Pol II, we removed DRB to release Pol II into the productive transcription stage, and labeled newly synthesized RNA with 4s-UTP at 5- and 10-min intervals, followed by purification and sequencing of labeled transcripts.

**Figure 4:**
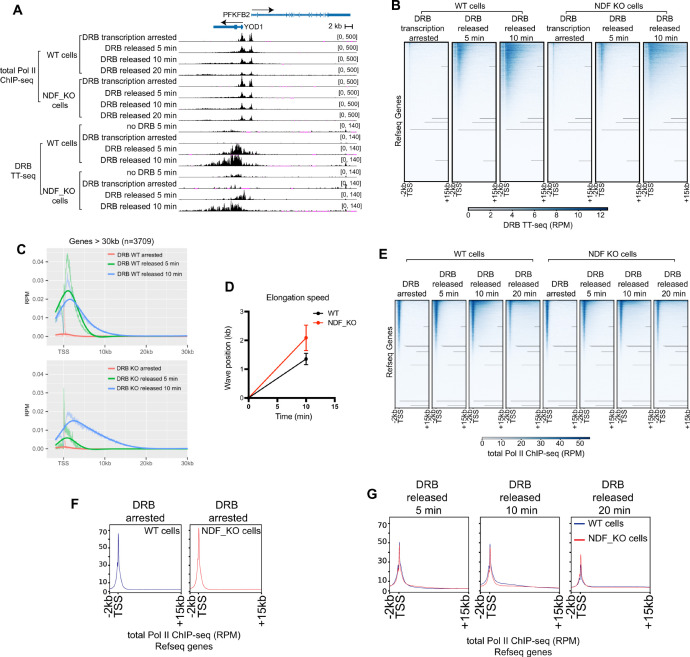
NDF is required for efficient transcription elongation processivity in cells. (**A**) Genome browser views comparing transcriptional activity between NDF wild-type (WT) and knockout (KO) HeLa cells at selected genomic loci. Cells were treated with the transcription inhibitor DRB for 3 h to synchronize transcription, followed by inhibitor release for the indicated times (5, 10, and 20 min). The upper tracks show Pol II ChIP-seq data reflecting Pol II occupancy, while the lower tracks show DRB/TT-seq data capturing nascent RNA synthesis. (**B**) Genome-wide heatmap analysis of nascent RNA levels from −2 kb to +15 kb relative to transcription start sites (TSS) in NDF WT and KO HeLa cells after DRB release for 5 and 10 min. Total RefSeq genes are sorted by average nascent RNA abundance (high to low). (**C**) Metagene analysis of DRB/TT-seq signal from non-overlapping genes (30–300 kb) on standard chromosomes (*n* = 3709), showing the average transcriptional wave progression after DRB release. Profiles extend from −2 kb to +30 kb relative to TSS. Solid lines represent computationally fitted splines. The wave front advances further in NDF KO cells despite producing less RNA, suggesting faster but less processive elongation. (**D**) Quantification of Pol II elongation rates was calculated by linear regression analysis of the transcriptional wave peak positions at 0 and 10 min after DRB release. Error bars represent standard deviation across the 3709 genes analyzed in this study from two independent experiments. (**E**) Heatmap representations of Pol II ChIP-seq data from −2 kb to +15 kb relative to TSS in NDF WT and KO cells after DRB release (0, 5, 10, and 20 min). (**F**) Expanded metagene profile of Pol II ChIP-seq data from −2 kb to +15 kb relative to TSS in NDF WT (blue) and KO (red) cells at 0 min (DRB arrest). (**G**) Expanded metagene profiles of Pol II ChIP-seq data from −2 kb to +15 kb relative to TSS in NDF WT (blue) and KO (red) cells at 5, 10, and 20 min after DRB release.

As shown in Figure 4A, genome browser views revealed abundant nascent transcripts downstream of promoter-proximal regions 5 min after DRB release in wild-type cells, with transcript waves extending further into gene bodies at 10 min as Pol II progressed. Genome-wide heatmap analysis revealed transcriptional waves progressing through gene bodies in both WT and NDF-KO cells (Figure 4B). However, when we quantified average elongation speed, the NDF-KO cells unexpectedly displayed similar or even faster elongation rates compared to wild-type cells, despite producing fewer transcripts overall (Figure 4C,D and Figure S2).

To determine whether reduced nascent transcript levels in NDF-KO cells stemmed from diminished processivity post-release, we investigated Pol II occupancy in gene bodies following DRB release (Figure 4A). Previous studies have established that transcription processivity can be measured by Pol II association across coding regions [61]. Using ChIP-seq to track total Pol II levels in a time-course experiment, we found that Pol II in NDF-KO cells initially elongated slightly faster than in wild-type cells, consistent with our DRB-TT-seq data.

During the first five minutes after DRB removal, both cell types showed comparable Pol II release from pausing sites, with NDF-KO cells exhibiting marginally higher release. However, by the 10-min timepoint, Pol II association with coding regions in NDF-KO cells was noticeably lower than in wild-type cells—a trend also observed at the 20-min timepoint (Figure 4E–G). This pattern indicates that while substantial amounts of Pol II were initially released from promoter-proximal pausing sites in both cell types, polymerases in NDF-KO cells could not maintain stable elongation between 5–10 min after release, resulting in reduced Pol II occupancy in gene bodies at later timepoints. We propose that without NDF, transcribing Pol II cannot stably associate with the DNA template, leading to premature termination, as evidenced by the decreased Pol II levels at 10- and 20-min timepoints. The 10-min timepoint provides the clearest evidence for this effect, as the 20-min timepoint may be influenced by polymerases that have completed a full round of transcription.

These results suggest that NDF primarily enhances transcription processivity in human cells. While Pol II in NDF-KO cells efficiently escapes promoter-proximal pausing and actually moves faster along the DNA template, it produces significantly fewer complete transcripts due to premature termination. This increased elongation rate may compensate for reduced processivity in cells under normal conditions (without DRB treatment), potentially explaining why mRNA levels remain largely unaffected in NDF knockout HeLa cells [44]. Thus, NDF’s critical function is maintaining stable Pol II-template interactions during elongation rather than regulating elongation speed.

### 2.7. NDF Mutants Affect Gene Expression in Cells

**Figure 5:**
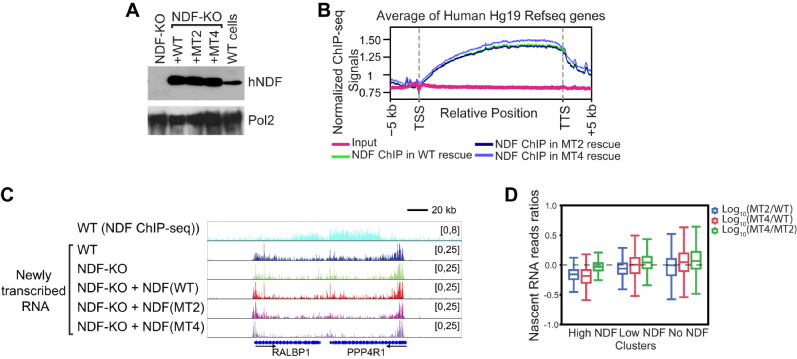
Mutations in the Pol II interaction domain of NDF impair transcription in cells. (**A**) Validation of the NDF rescue system in SW480 colon cancer cells. Western blot analysis confirms comparable expression levels of wild-type (WT) hNDF and interaction domain mutants (MT2 and MT4) in NDF knockout (KO) cells, with the Rpb1 subunit of Pol II serving as a loading control. Some lanes were cropped and previously published in Fei et al., *Genes Dev*, 2022 [44]. (**B**) Metagene analysis of NDF genomic occupancy as determined by ChIP-seq in WT, MT2, and MT4 rescued SW480 cells. The profiles show NDF distribution across gene bodies. MT2 and MT4 mutants retain normal genomic localization despite their impaired Pol II interaction, indicating that NDF recruitment to chromatin is independent of its ability to stimulate Pol II. (**C**) Representative genome browser views comparing NDF ChIP-seq signal in WT cells with nascent RNA-seq profiles in WT, KO, and mutant-rescued cell lines at selected genomic loci. (**D**) Quantitative analysis of nascent RNA production across different gene categories based on NDF occupancy levels. Genes were clustered into High NDF, Low NDF, and No NDF groups based on ChIP-seq signal intensity. The analysis reveals that MT2 and MT4 mutants fail to rescue transcriptional defects in the High NDF gene cluster.

Having established NDF’s function in transcription elongation in cells, we investigated whether the interaction between NDF and Pol II is functionally significant in cells. We rescued hNDF knockout (KO) human SW480 colon cancer cells with wild-type hNDF, MT2, or MT4. Western blot analysis confirmed comparable expression levels of wild-type and mutant NDF proteins (Figure 5A).

To determine whether these mutations affect genomic localization, we performed NDF ChIP-seq assays. Metagene analysis of the ChIP-seq data revealed that MT2 and MT4 were enriched in the gene body region, similar to wild-type NDF, suggesting that the mutations do not alter the genomic localization of hNDF proteins (Figure 5B). To evaluate the impact of NDF on transcription, we measured nascent RNA production using Br-UTP labeling, which allows detection of newly synthesized transcripts by incorporating bromouridine into RNA during a short pulse in living cells [62]. Following immunoprecipitation of Br-UTP-containing RNA and deep sequencing, we classified genes based on their NDF occupancy as determined by ChIP-seq [42]. While NDF knockout strongly reduced nascent RNA production from genes with high NDF occupancy, wild-type NDF expression successfully rescued this transcription defect [42]. Importantly, cells expressing MT2 or MT4 mutants failed to rescue the reduced nascent transcript phenotype in high-NDF genes (Figure 5C,D). This deficiency was consistently observed across biological replicates, demonstrating that the NDF-Pol II interaction interface is essential for NDF’s function in stimulating transcription elongation in cells (Supplementary Figure S3). Quantitative analysis further confirmed that most high-NDF genes showed significantly reduced transcription when MT2 or MT4 were expressed compared to wild-type NDF. Taken together, these findings suggest that the specific interaction between NDF and Pol II is crucial for effective Pol II stimulation in cells.

## 3. Discussion

Our findings reveal that NDF employs dual mechanisms to stimulate transcription elongation through functionally distinct domains. Through comprehensive biochemical and mutational analysis, we identified a critical region (amino acids 140–160) that specifically interacts with the hyperphosphorylated Rpb1 subunit of elongating RNA Polymerase II. This interaction domain functions independently from NDF’s previously established nucleosome-destabilizing activity, as evidenced by our mutant MT2, which abolished Pol II stimulation while fully preserving nucleosome destabilization. These results provide strong evidence that NDF contains modular domains that independently contribute to transcription elongation, directly stimulating Pol II activity even on naked DNA templates while also modifying chromatin accessibility through a separate function.

Mechanistically, our findings suggest a dynamic interplay between NDF’s functions. NDF’s conserved PWWP domain, known to recognize histone H3K36me3 marks, appears to also participate in binding elongating Pol II, together with the central intrinsically disordered region we identified. This implies a potential “handoff” model where NDF is initially recruited via Pol II and then transferred to chromatin as the polymerase progresses. In this scenario, the PWWP domain would relinquish Pol II and engage H3K36me3 on nucleosomes once they become modified during transcription, positioning NDF to loosen nucleosomes at precisely the right time and location.

The biological implications of NDF extend beyond basic transcription mechanisms to organ development and disease. A particularly significant example is in cardiac development, where NDF/GLYR1 directly interacts with the master cardiac transcription factor GATA4. Loss-of-function mutations in GLYR1 disrupt this interaction, leading to reduced expression of GATA4 target genes and resulting in congenital heart defects [63]. This finding directly links NDF’s elongation function to the activation of critical gene networks during organogenesis, without NDF, even initiated transcripts may not efficiently elongate to produce necessary RNA and protein levels during critical windows of heart morphogenesis. Beyond development, NDF is also frequently overexpressed in cancers [40], particularly breast carcinoma, suggesting that tumor cells may leverage NDF’s dual functionality to drive oncogenic transcription programs.

Comparisons with other elongation factors highlight NDF’s unique mode of action. The histone chaperone FACT, for instance, facilitates Pol II passage through chromatin but does so without direct interaction with Pol II or DNA [22,27,64,65]. FACT primarily recognizes partially disassembled nucleosome structures [21,22,27]. It acts by removing or repositioning histones (notably the H2A–H2B dimer) once the DNA has transiently peeled away, thereby allowing Pol II to traverse and subsequently helping to reassemble the nucleosome [66]. NDF, in contrast, actively binds both the nucleosome (via the PWWP domain) and Pol II (via its central region), implying that it can initiate nucleosome destabilization and simultaneously ride along with the polymerase. We speculate that NDF and FACT may function in tandem: NDF could trigger nucleosome loosening and propel Pol II forward, while FACT follows to manage histone eviction and redeposition. Such cooperation would combine NDF’s chromatin-destabilizing impetus with FACT’s chaperone activity, ensuring both efficient transcript elongation and preservation of chromatin integrity.

In summary, NDF emerges as a multifaceted elongation factor that coordinates chromatin remodeling and Pol II stimulation through distinct functional domains. Moving forward, high-resolution structural analyses, particularly using advanced cryo-EM techniques, will be essential to precisely define how NDF structurally engages Pol II, LSD2, and chromatin, further clarifying its unique contribution to transcription elongation control.

## 4. Materials and Methods

### 4.1. Cell Culture

SW480, HeLa, and HEK293T cells were maintained in DMEM (Corning, Corning, NY, USA) with 10% FBS (Corning) and 100 U/mL penicillin-streptomycin (ThermoFisher, Waltham, MA, USA). Cells were maintained in a humidified incubator atmosphere at 37 °C with 5% CO_2_. NDF knockout (KO) SW480 cells and WT rescued cells were from the previous study [42].

### 4.2. Antibodies

Rabbit polyclonal antisera against hNDF were generated as previously described [44]. Commercial antibodies used included anti-Rpb1 NTD (Cell Signaling, Danvers, MA, USA, #14958) and anti-Rpb2/Pol II (Genetex, Irvine, CA, USA, #GTX102535).

### 4.3. Plasmid Construction

Recombinant hNDF-expressing vectors pET21b-His6-hNDF for bacterial expression were cloned as described previously. For hNDF truncates expressing vectors, the corresponding DNA fragments were subcloned similarly to the full-length hNDF. The BirA* construct (a gift from Enfu Hui’s lab, UCSD) was inserted into an NDF-His6-containing plasmid using Gibson assembly (NEB) to generate the pET21b-N-His6-BirA*-hNDF expression construct. Expression vectors for NDF mutants were generated with a Q5 site-directed mutagenesis kit (NEB). using the following primers: for F1AM and MT1 mutants, primers NDF-F1A-For (5′- GCCGCCGCCGCCGCCAGGGTGTCTTCAGGCTCTTCAG-3′) and NDF-F1A-Rev (5′- CATGTTCTTCTTCACCTTCCC-3′) were used; for F1BM and MT2 mutants, primers NDF-F1B-For (5′-GCCGCCGCCGCCGCCTCTTCAGAGAGAGGCTCCAAATC-3′) and NDF-F1B-Rev (5′-CTTCTTTCCTTCTCCCATGTTC-3′) were used; for F1CM and MT3 mutants, primers NDF-F1C-For (5′-GCCGCCGCCGCCGCCTCCAAATCCCCTCTGAAAAGAGC-3′) and NDF-F1C-Rev (5′-GCCTGAAGACACCCTCTTCTTTC-3′) were used; for F1DM and MT4 mutants, primers NDF-F1D-For (5′-GCCGCCGCCGCCGCCAAAAGAGCCCAAGAGCAAAGTC-3′) and NDF-F1D -Rev (5′-GCCTCTCTCTGAAGAGCCTGAAG-3′) were used; All expression constructs were sequenced for verification.

### 4.4. Protein Purification

Human NDF WT, truncates, mutants, and hNDF-BirA recombinant proteins were purified as previously described [44]. The *S. cerevisiae* RNA Polymerase II (Pol II) and human Pol II were purified as described [42].

### 4.5. Pull-Down Assay

For the in vitro pull-down assay, unless specified, 40 pmol of His6-tagged NDF WT, truncates, or mutants were incubated with 20 pmol of yeast or human Pol II proteins and 40 µL Ni-NTA beads in 300 μL of pull-down buffer (10 mM Tris-HCl [pH 7.5], 200 mM NaCl, 0.2% Nonidet P-40, 10% glycerol, Protease Inhibitor Cocktail [Roche]) at 4 °C for 3 h. Then the beads were captured by centrifugation for 1 min at 1000 g and washed three times with the same pull-down buffer. Proteins were eluted with SDS-PAGE buffer and visualized by SDS-PAGE and silver staining or Western blot.

### 4.6. Ndf Knockout and Knockout Rescue in Sw480 Cells

SW480 KO cells expressing hNDF-MT2 and hNDF-MT4 were produced as previously described [2]. HEK293T cells were transfected with pHR-IRES-puro-NDF-MT2 or MT4 vector, psPAX2 (addgene #12260), and pMD2.G (addgene #12259) to generate lentivirus. At 48 h post-transfection, the media from HEK293T cells, which contain the lentiviruses, was harvested and used to infect SW480 KO cells. Stable cells were established by selecting with 1 µg/mL puromycin.

### 4.7. In Vitro Biotinylation Assay

For the in vitro biotinylation assay, reactions were performed in a final volume of 20 μL using increasing amounts of hNDF-BirA* (0, 1, 2, 4, and 8 pmol) incubated with 25 pmol yPol II in biotinylation buffer (20 mM HEPES, pH 7.5, 10 mM MgCl_2_, 100 mM KCl, 1.5 mM ATP, and 5% glycerol). Biotin was added to a final concentration of 0.5 μM to initiate the reaction, followed by incubation at 30 °C for 1 h. The reactions were then stopped by adding an equal volume of 2× SDS-PAGE loading buffer, and biotinylated proteins were separated by SDS-PAGE gel and further detected by Western blotting.

### 4.8. Western Blot

Cells were lysed in 1X SDS sample buffer containing 50 mM Tris-HCl (pH 6.8), 1% SDS, 8% glycerol, 0.02% bromophenol blue, and 2% 2-mercaptoethanol. The lysates were briefly sonicated and heated at 95 °C for 5 min to denature proteins. Protein separation was performed using SDS-PAGE. After incubation with primary antibodies (as described above), the membrane was treated with HRP-conjugated Protein A (Cell Signaling 12291). For detecting biotinylated proteins, the membrane was directly incubated with HRP-conjugated streptavidin. Detection was carried out using SuperSignal West Pico PLUS Chemiluminescent Substrate (ThermoFisher), and the signal was captured with a ChemiDoc Imaging System (Bio-Rad).

### 4.9. In Vitro Transcription Assays with Calf Thymus DNA

In vitro transcription assays with calf thymus DNA were performed as previously described [42]. The calf thymus DNA (Sigma-Aldrich, St. Louis, MO, USA; D1501; 100 mg) was extracted three times with phenol-chloroform-isoamyl alcohol (25:24:1, *v*/*v*/*v*), and the final DNA was resuspended in TE to a final concentration of 1 mg/mL. The transcription reactions were performed in a final volume of 20 μL, consisting of 9 μL of TE’ buffer (10 mM Tris-HCl, pH 8.0, 0.1 mM EDTA), 2 μL of 10× Transcription Buffer (200 mM Tris-HCl, pH 7.5, 50 mM MgCl_2_, 400 mM KCl, 100 mM DTT), 1 μL of 10 mM ATP, UTP, GTP, 2 μL of 1 mg/mL calf thymus DNA, 2 μL of 75 nM Pol II or its diluent, and 2 μL of hNDF buffer (10 mM Tris-HCl, pH 7.5, 0.2% Nonidet P-40 (Pierce), 0.2 M NaCl, 10% glycerol, 250 mM imidazole, and 5 mM 2-mercaptoethanol), along with 2 μL 1 μM hNDF WT or mutants. The reaction mixture was incubated at 30 °C for 10 min, followed by the addition of 2 μL of 1 mM [α-^32^P]CTP (700–1000 cpm/pmol) and further incubation at 30 °C for 30 min. To terminate the reactions, the samples were spotted onto 2.4 cm DE81 filter disks (Whatman, Marlborough, MA, USA) and allowed to dry for approximately 10 min. The filters were then washed in 100 mL of 0.35 M Na_2_HPO_4_ for 5 min with occasional swirling. This phosphate buffer exchange was repeated 6 to 7 times, followed by two washes with 100 mL of H_2_O and a final wash with 100% ethanol. The filters were air-dried for at least 15 min before being subjected to scintillation counting.

### 4.10. Transcription Elongation Assays

The transcription elongation assays were performed as previously described [2]. In brief, the 10-nucleotide 5′-³²P-labeled RNA primer was annealed to an equimolar amount of template strand (TS) DNA. Purified human Pol II was then added to the RNA-TS complex, and the mixture was incubated at 30 °C for 10 min. Subsequently, the 5′-biotin-labeled non-template strand (NTS) was introduced and incubated at 30 °C for 15 min to assemble the elongation complex, which was then immobilized onto Dynabeads MyOne Streptavidin C1 magnetic beads (Invitrogen). Following immobilization, the beads were washed and ligated to the downstream 5S DNA fragment. The elongation complexes were then distributed into individual tubes, where hNDF WT, its mutants, or the corresponding buffer (negative control) was added. Transcription elongation was initiated by the addition of rNTPs, and reactions proceeded at 22 °C for the specified durations. To terminate the reactions, an equal volume of stopping buffer (1 mL of gel loading buffer consisting of 0.9 mL deionized formamide, 0.1 mL 0.5 M EDTA, and 2 μL of 4% [*w/v*] bromophenol blue) was added. Samples were then analyzed by denaturing 8% polyacrylamide-urea gel electrophoresis. The resulting gel images were acquired using the Amersham™ Typhoon (Marlborough, MA, USA) imaging system. The sequences of the template strand (TS), non-template strand (NTS), RNA primer, and 5S rDNA were identical to those described in Fei et al. [44].

### 4.11. DNA Supercoiling Assays

DNA supercoiling assays were performed as previously described [44]. Briefly, a master mix was prepared containing 0.3 μM nucleosomes (reconstituted onto plasmid DNA by salt dialysis), 0.1 units topoisomerase I, and 1.4 μg BSA (NEB) in buffer S (25 mM Hepes, K+, pH 7.6, 100 mM KCl, 0.2 mM MgCl_2_, 0.1 mM EDTA, and 10% (*v*/*v*) glycerol). This master mix was aliquoted into individual tubes before adding different concentrations of hNDF WT or mutants (50 μL final volume each). The mixture was incubated at 30 °C for 1 h, followed by deproteinization with proteinase K. Then, the plasmid DNA was extracted with phenol-chloroform-isoamyl alcohol (25:24:1, *v*/*v*/*v*), and the recovered DNA was visualized with 0.8% agarose gel electrophoresis. Nucleosome destabilization activity was quantified using ImageJ software (Version 2.16.0/1.54p) [67]. For each lane, the total intensity of supercoiled, nicked, and relaxed DNA species was measured. The percentage of each species was calculated by normalizing to the total DNA intensity in that lane. Background activity was determined from the buffer-only control (polynucleosomes + topoisomerase I without NDF), and this background percentage was subtracted from all experimental conditions. The resulting relaxed DNA percentage for wild-type NDF at 1× concentration was set as the reference (activity = 1.0), and all other conditions were normalized to this value.

### 4.12. ChIP-Seq Analysis

The ChIP-Seq was performed as described [44] with slight modifications. 10–15 million SW480 NDF KO with hNDF WT or mutant rescued cells were collected and crosslinked with 1% formaldehyde (Sigma) for 10 min at 37 °C. Then, resuspend the crosslinked cell pellet in 5 mL cell Lysis Buffer (5 mM Hepes, K+, pH 8.0, 85 mM KCl, 0.5% [*v*/*v*] Nonidet P-40, and Protease Inhibitor Cocktail (Roche)) and incubate on ice for 5 min. After incubation, pellet the cells by centrifuge at 500 g at 4 °C for 5 min, then the pellet was resuspended in 1 mL nuclear lysis buffer (50 mM Tris-HCl, pH 8.0, 10 mM EDTA, 0.2% [*w*/*v*] SDS, and Protease Inhibitor Cocktail (Roche)) and sonicated with a Covaris S220 sonicator (Woburn, MA, USA). Chromatin concentrations were estimated using a Nanodrop spectrophotometer (ThermoFisher) and diluted to a final concentration of 0.5 mg/mL with ChIP dilution buffer (16.7 mM Tris-HCl, pH 8.0, 167 mM NaCl, 1.2 mM EDTA, 0.01% [*w*/*v*] SDS, 1.1% [*v*/*v*] Triton X-100, and Protease Inhibitor Cocktail (Roche)). 80% glycerol was added to the chromatin sample to make it 0.5 mg/mL with 10% glycerol. For immunoprecipitation, 0.15 mg chromatin and 200 μL of ChIP dilution buffer were added to 20 μL of Dynabeads Protein A (ThermoFisher) pre-bound with specific antibodies (1 μL hNDF antiserum for NDF ChIP, 4 μL RPB II antibody for total Pol II ChIP) and incubated at 4 °C for 4 h on a nutator. Beads were captured with a magnetic stand and washed as described [44]. Chromatin bound to the beads was eluted twice with 50 μL of TE (10 mM Tris-HCl, pH 8, 1 mM EDTA) containing 1% [*w*/*v*] SDS for 20 min at 20°C. ChIP-seq libraries were prepared with the NEBNext Ultra II DNA Library Prep Kit for Illumina (NEB) according to kit instructions. Final libraries were quantified by a Qubit 4 fluorometer and sequenced with an Illumina HiSeq 4000 sequencer (San Diego, CA, USA, UCSD sequencing core). The sequencing data were processed using the usegalaxy.org server [68]. Raw reads were trimmed with Fastp [69] using default settings and further processed with Trim Galore! [70] using automatic adapter detection and trimming. Trimmed reads were aligned to the hg19 reference genome using Bowtie2 [71] with local alignment mode. PCR duplicates were removed using Samtools with default parameters [72]. For data visualization, bigwig files were generated using Homer [73] makeTagDirectory command, followed by conversion of bedGraph files to bigwig format, with normalization to total mapped reads. Metagene analysis was performed using deepTools [74]: the computeMatrix command was used to generate the analysis files, and plotHeatmap was used to create the metagene plots and heatmaps.

### 4.13. TT-Seq and BRU-Seq

TT-seq was performed as previously described [75,76], with modifications. For each biological replicate, 10 million cells were seeded on one 15 cm dish one day before the experiment. On the experiment day, cells were approximately 80% confluent. Cells were treated with 0.1 mM DRB (TCI) for 3 h prior to the experiment. For 4-thiouridine (4sU, Sigma) labeling, 4sU was added to a final concentration of 1 mM in culture media. After DRB-arrested cells were quickly washed twice with warm 1× PBS, 4sU-containing culture media was added to the cells and incubated for the indicated times at 37 °C in the incubator. Once labeling was completed, cells were quickly washed with 1× cold PBS, residual liquid was quickly removed from the culture plate, and 3 mL Trizol LS (ThermoFisher) was added directly to the cells. RNA was purified twice using a standard Trizol RNA extraction protocol. Residual DNA was removed by treating the RNA sample with DNase I, followed by repurification using Trizol. Biotinylation of 4sU-labeled RNA was performed as previously described [76]. Briefly, 50 µL of 1 mg/mL Biotin-HPDP (ThermoFisher) was added to 100 µL of RNA sample, followed by the addition of 100 µL of 2× labeling buffer (25 mM Tris-HCl, pH 7.5, and 2.5 mM EDTA). Since Biotin-HPDP has limited solubility in aqueous solutions, the reaction may appear cloudy; in this case, 20 µL of DMSO was added to improve Biotin-HPDP solubility. The reaction was incubated at room temperature for 2 h with gentle rotation on a nutator. Samples were subsequently purified using Trizol and resuspended in H_2_O. Biotinylated RNA was briefly heat-denatured and then isolated using 50 µL of M2 Streptavidin beads (ThermoFisher). Purified RNA was fragmented using RNA fragmentation reagents (ThermoFisher). Bru-seq was performed as previously described [42,62]. RNA libraries were prepared with the NEBNext Small RNA Library Prep Set (NEB) and sequenced on a HiSeq 4000 sequencer (Illumina) at the UCSD sequencing core. Raw sequencing data were trimmed and aligned to the hg19 reference genome using Bowtie2, and PCR duplicates were removed with samtools. The mapped BAM files were used for analyzing the wave peak and elongation speed as previously described with slight modifications [77]. Calculations were restricted to genes > 30 kb with non-overlapping transcription units (corresponding to 3709 hg19 transcripts), and gene ranges were extended around their TSSs (−2 kb to +30 kb); extensions beyond chromosome limits were dropped.

The genome-wide sequencing data have been deposited in the Gene Expression Omnibus database and will be made publicly accessible upon manuscript acceptance.

## Data Availability

Genome sequencing results are available on GEO under accession number GSE294087 and GSE294088.

## Supplementary Materials

The following supporting information can be downloaded at: https://www.mdpi.com/article/10.3390/ijms26104874/s1.
