# Supplementary material for: NDF/GLYR1 Promotes RNA Polymerase II Processivity via Pol II Binding and Nucleosome Destabilization
Source: Int J Mol Sci. 2025 May 19;26(10):4874. doi: 10.3390/ijms26104874 (PMC12112590; doi:10.3390/ijms26104874)
Supplement: Supplementary file 1 [file ijms-26-04874-s001.zip › ijms-3603064-supplementary.pdf]

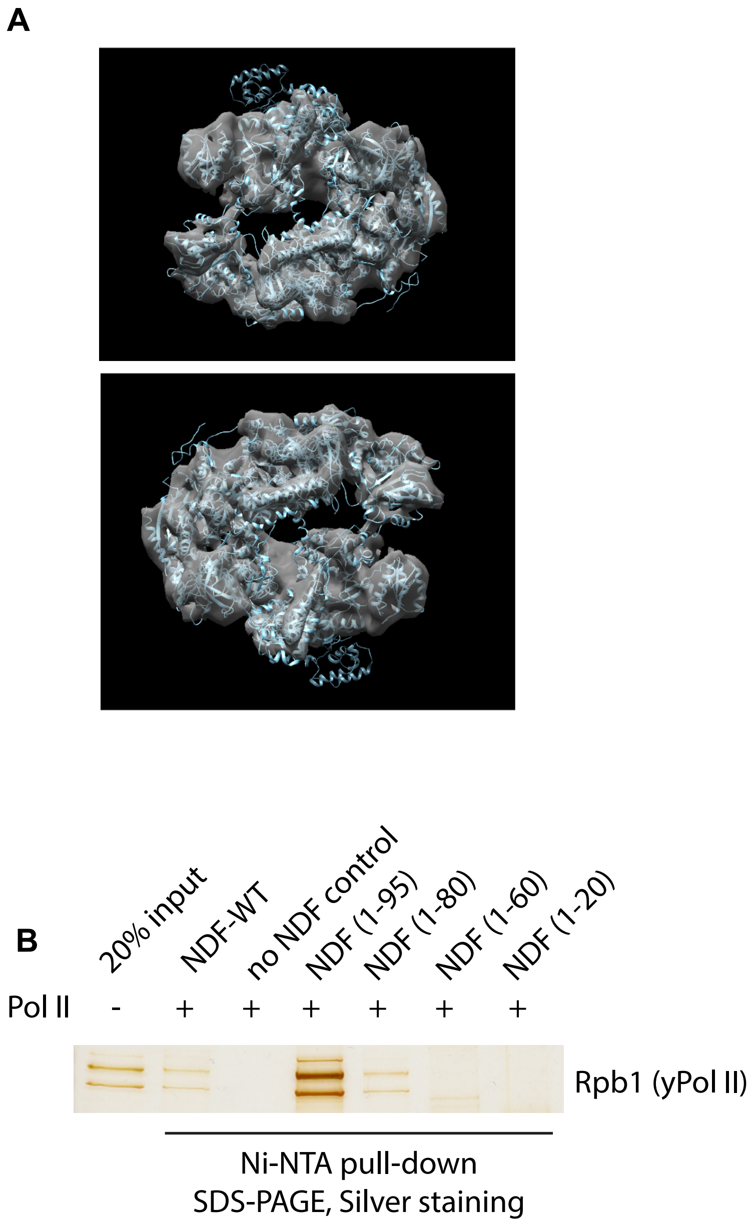

Supplemental Figure S1.

A. Cryo-EM characterization of the NDF-yPol II complex. Electron density map of the glutaraldehyde crosslinked NDF-yeast Pol II complex following purification by Superose 6 size exclusion chromatography. Complex formation was previously confirmed by SDS-PAGE and silver staining as described in Fei et al. Genes Dev, 2022. The electron density map was obtained from processing ~4,000 collected micrographs at the Rutgers Cryo-EM Core Facility. The map is overlaid with the human RNA Pol II cryo-EM structure (PDB: 6DRD) shown in gray ribbon representation. No additional electron density corresponding to the NDF protein was observed under the conditions used.

B. Mapping the Pol II interaction region within the PWWP domain of NDF.

Pull-down assays identifying the critical region in the PWWP domain responsible for Pol II interaction. His-tagged NDF constructs containing various deletions were immobilized on Ni-NTA agarose beads and incubated with purified yeast Pol II. Bound proteins were analyzed by SDS-PAGE followed by silver staining. Some NDF constructs stained poorly with silver staining but were verified by Coomassie staining (not shown). The results indicate that amino acids 60-95 of human NDF are critical for the interaction with Pol II.

A

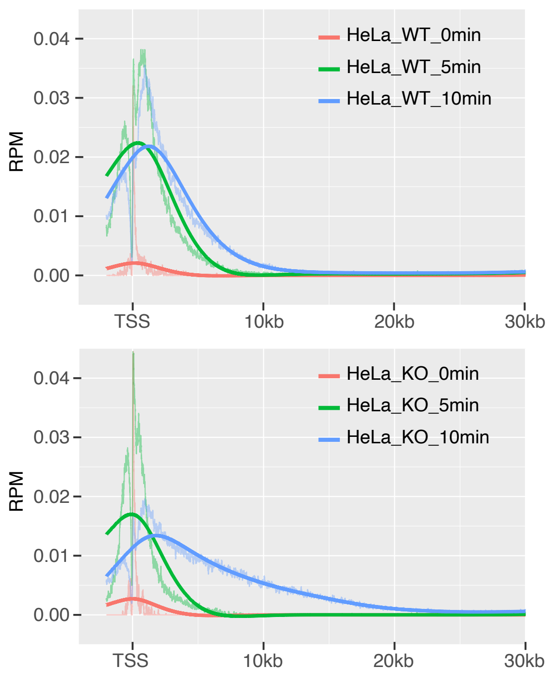

Supplemental Figure S2.
A, Metagene analysis of DRB/TT-seq signal from non-overlapping genes (30-300kb) on standard chromosomes (n=3,709) from a biological duplicate experiment, showing the average transcriptional wave progression after DRB release. Profiles extend from -2kb to +30kb relative to TSS. Solid lines represent computationally fitted splines. The wave front advances further in NDF KO cells despite producing less RNA, suggesting faster but less processive elongation.

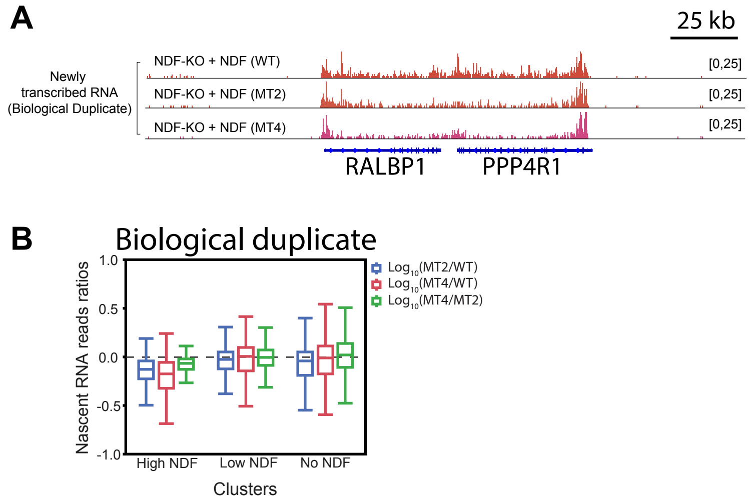

Supplemental Figure S3.
A, Representative genome browser views comparing NDF ChIP-seq signal in WT cells with nascent RNA-seq profiles in WT, KO, and mutant-rescued cell lines at selected genomic loci from a biological duplicate experiment.
B, Quantitative analysis of nascent RNA production across different gene categories based on NDF occupancy levels from a biological duplicate experiment. Genes were clustered into High NDF, Low NDF, and No NDF groups based on ChIP-seq signal intensity. The analysis reveals that MT2 and MT4 mutants fail to rescue transcriptional defects in the High NDF gene cluster.
